# HLA-DR7 and HLA-DQ2: Transgenic mouse strains tested as a model system for ximelagatran hepatotoxicity

**DOI:** 10.1371/journal.pone.0184744

**Published:** 2017-09-21

**Authors:** Hanna Lundgren, Klara Martinsson, Karin Cederbrant, Johan Jirholt, Daniel Mucs, Katja Madeyski-Bengtson, Said Havarinasab, Per Hultman

**Affiliations:** 1 Division of Molecular and Immunological Pathology, Department of Clinical and Experimental Medicine, Faculty of Health Sciences, Linköping University, Östergötland County Council, Linköping, Sweden; 2 AIR/Rheumatology Unit, Department of Clinical and Experimental Medicine, Faculty of Health Sciences, Linköping University, Östergötland County Council, Linköping, Sweden; 3 Swetox, Karolinska Institutet, Unit of Toxicology Sciences, Södertälje, Sweden; 4 AstraZeneca R&D, Transgenic center, Mölndal, Sweden; Medizinische Fakultat der RWTH Aachen, GERMANY

## Abstract

The oral thrombin inhibitor ximelagatran was withdrawn in the late clinical trial phase because it adversely affected the liver. In approximately 8% of treated patients, drug-induced liver injury (DILI) was expressed as transient alanine transaminase (ALT) elevations. No evidence of DILI had been revealed in the pre-clinical *in vivo* studies. A whole genome scan study performed on the clinical study material identified a strong genetic association between the major histocompatibility complex alleles for human leucocyte antigens (HLA) (HLA-DR7 and HLA-DQ2) and elevated ALT levels in treated patients. An immune-mediated pathogenesis was suggested. Here, we evaluated whether HLA transgenic mice models could be used to investigate whether the expression of relevant HLA molecules was enough to reproduce the DILI effects in humans. In silico modelling performed in this study revealed association of both ximelagatran (pro-drug) and melagatran (active drug) to the antigen-presenting groove of the homology modelled HLA-DR7 molecule suggesting “altered repertoire” as a key initiating event driving development of DILI in humans. Transgenic mouse strains (tgms) expressing HLA of serotype HLA-DR7 (HLA-DRB1*0701, -DRA*0102), and HLA-DQ2 (HLA-DQB1*0202,–DQA1*0201) were created. These two lines were crossed with a human (h)CD4 transgenic line, generating the two tgms DR7xhCD4 and DQ2xhCD4. To investigate whether the DILI effects observed in humans could be reproduced in tgms, the mice were treated for 28 days with ximelagatran. Results revealed no signs of DILI when biomarkers for liver toxicity were measured and histopathology was evaluated. In the ximelagatran case, presence of relevant HLA-expression in a pre-clinical model did not fulfil the prerequisite for reproducing DILI observed in patients. Nonetheless, for the first time an HLA-transgenic mouse model has been investigated for use in HLA-associated DILI induced by a low molecular weight compound. This study shows that mimicking of genetic susceptibility, expressed as DILI-associated HLA-types in mice, is not sufficient for reproducing the complex pathogenesis leading to DILI in man.

## Introduction

Attritions in late drug development phase are extremely costly and one of the most unwanted outcomes for pharmaceutical companies. Drug-induced liver injury (DILI) is a major cause for pharmaceuticals being withdrawn post marketing [1] and belongs to one of the most common safety issues in preclinical studies [2]. Ximelagatran, a direct thrombin inhibitor marketed as Exanta^™^ and launched in Europe in 2004 [3], was developed for the prevention and treatment of thromboembolic conditions. In long-term clinical studies (>35 days) [4], almost 8% of the ximelagatran treated patients had an elevated alanine transaminase (ALT) of >3 times the upper limit of normal (ULN), a level typically observed within the first six months of dosing [5,6]. Elevated ALT levels were also detected after short-term treatment (<35 days), and ximelagatran was withdrawn in 2006 [7,8]. No signs of hepatotoxicity could be revealed during the regular development program nor later in the extended mechanistic investigative *in vitro* studies [9] and *in vivo* studies in mouse, rat, dog, guinea-pig, and cynomolgus monkey [10].

To trace possible adverse outcome pathways (AOPs) and to find relevant biomarkers used to exclude patients at risk, a number of problem-solving studies (genomics, metabolomics, proteomics transcriptomics, ligand fishing, and more) were initiated. Most interestingly, a whole genome scan study [11] showed significant correlations between increased levels of ALT and presence of the highly variable major histocompatibility complex (MHC) class II alleles human leucocyte antigen (HLA)-DRB1*07 (odds ratio 4.4 [95% CI 2.2–8.9]) and HLA-DQA1*02 (odds ratio 4.4 [CI 2.2–8.8]) in affected patients. Expressed in 15% of European Americans [12], HLA-DRB1*07 and HLA-DQA1*02 are strongly linked and practically co-inherited. The available data [11] reveal that the two alleles appear to be equally important. Associations between genetic sequences and either efficacy or safety of drugs at odds ratios >3.0 (equivalent to >300% increased efficacy or safety) has been suggested to be useful in clinical practice [13,14].

Recently, a growing number of HLA-linked adverse drug reactions that involve small molecule drugs have been published [15–19]. Finding associations between specific HLA alleles and increased susceptibility to DILI, Flucloxacillin [20,21] and Diclofenac [22], and the possibility of using HLA-typing in risk management, e.g., HLA-B*57:01 for avoiding Abacavir hypersensitivity [15,23–25], are important steps forward in understanding the key events leading to immune-mediated adverse drug-reactions. The haplotype of interest–HLA-DQA1*02-HLA-DRB1*0701 –has been associated with DILI [26,27]. Our hypothesis involves ximelagatran acting as an inducer of immune-mediated DILI driven by the “altered repertoire” hypothesis [28] and in silico-modelling has been used to investigate possible drug-associations with DR7.

This study generates a new pre-clinical model using low molecular weight (LMW) drug development where drug recognition by specific HLA-alleles is the key initiating event and AOP leading to an immune-mediated adverse drug effect. We constructed two transgenic mouse strains (tgms)–DR7 (HLA-DRB1*0701, -DRA*0102) and DQ2 (HLA-DQB1*0202, DQA1*0201)–that were further crossed with mice expressing human (h)CD4 to create two double tgms: DR7xhCD4 and DQ2xhCD4. These strains were then characterized by immune phenotypic and functional tests and subsequently exposed orally to ximelagatran in a 28-day repeated-dose study.

The high-mobility group protein B1 (HMGB1), used as an inflammatory and necrosis indicator *in vitro* [29,30] and as a hepatotoxicity marker in humans [31], was included as a biomarker for hepatotoxicity together with soluble colony-stimulating factor 1 receptor (CSF1R). Elevated plasma levels of CSF1R were earlier detected in patients with ALT-elevations after ximelagatran treatment [32]. Glutamate dehydrogenase (GLDH) was also analyzed as yet another hepatotoxicity marker since it has a higher sensitivity and specificity than ALT [33]. In summary, the biomarkers HMGB1, CSF1R, GLDH and ALT, together with histopathological evaluations, were used to look for possible translation between DILI in humans and a potential DILI in HLA-expressing tgms after ximelagatran exposure.

## Materials and methods

### Animals

During all studies, male and female mice between 7- and 16-weeks old were tested, bred, and maintained in the animal department at AstraZeneca R&D in Södertälje and Mölndal, Sweden. The animal experiments were approved by Stockholm South region ethics committee (Sweden). Animals were multiple-housed under pathogen-free conditions, observed once or twice daily, fed standard chow (RM1 (E) SQC pelleted, Special Diets Services Ltd., England), and provided tap water *ad libitum*. Blood samples were taken and final bleeding made under isoflurane anesthesia. No significant difference in mean weight between groups could be seen during any phase of the study. The wild type (wt) strain used was C57Bl/6NCrl.

### Generation of tgms

We generated two separate double transgenic mouse lines expressing the human MHC of serotypes HLA-DR7 and HLA-DQ2. The transgenic mouse lines were built to express the MHC class II α and β cDNAs, each under the control of a separate mouse H2-Eα promoter and followed by a β-globin poly adenylation signal sequence, both from the plasmid pDOI5 (kindly provided by Dr. Benoist) [34]. The HLA-DR7 transgene expressed the alleles HLA-DRA1*0102 and HLA-DRB1*0701, and the HLA-DQ2 transgene expressed the alleles HLA-DQA1*0201 and HLA-DQB1*0202. To minimize dysregulation and/or integration effects, each expression unit was flanked by double 1.2 kb insulator, a CTCF binding site derived from the chicken beta globin locus [35]. Both of these mouse lines were subsequently crossed onto a hCD4 expressing mouse line. The hCD4 expressing mouse was produced through random integration of a 42.5kb fragment of a human BAC clone (RPCI-11 101F21), resulting in the insertion of the fragment Chr12:6888111–6930612 (according to the GRCh37/hg19 assembly). The fragment contains 21.2 kb sequence upstream of the translational start and the human CD4 exon and intron structure, including the untranslated regions. After crossing the lines, two double transgenic mouse lines–DR7xhCD4 and DQ2xhCD4 –were produced.

### Characterization of tgms

#### Genotyping

Genotypes were determined by PCR amplification of genomic DNA derived from mouse ear biopsies using the following primers: H2Ea Forw; 5’-ATTCTGGCTGGCGTGGAAAT-3’, DQA Rev; 5’-AGACAGATGAGGGTGTTGGG-3’, DQB Rev; 5’-CTGGAAGGTCCAGTCACCAT-3’, DRA Rev; 5’-AGCATCAAACTCCCAGTGCT-3’, DRB Rev; 5’-TGTCCTCCAGGATGTCCTTC-3’, CD4 Forw; 5’-GCACCACTTTCTTTCCCTGA-3’ and CD4 Rev; 5’-CCCAGCCTAGTATATGCCCA-3’. The PCR products were run on a 0.8% agarose gel. Animals were used as heterozygotes for all transgenic (tg) constructs.

#### Phenotyping and verification of HLA and hCD4 expression

Flow cytometric (FACS) immunophenotyping and control of the expression of HLA-DR7, HLA-DQ2, and hCD4 was performed on peripheral blood mononuclear cells (PBMC) and spleen cells from tgms, DR7xhCD4 (n = 5), DQ2xhCD4 (n = 2), and wt (n = 5) animals. For the FACS analysis, spleens were collected and transferred to RPMI 1640 medium (Gibco) supplied with 10mM HEPES, 4mM L-glutamine, and 10% fetal calf serum (Gibco). Spleens were single cell suspended using Medimachine^™^ (BD Biosciences), washed, and labelled with antibodies according to manufacturer’s instructions. Blood samples were treated with FACS^™^ lysing solution (BD, USA) and incubated for five minutes at room temperature (RT) followed by antibody labelling. The following mAbs were used: anti-mouse (m)CD3, anti-mCD4, anti-mCD8a, anti-mCD19, anti-mCD49b, anti-human (h)HLA-DR (clone: G46-6 (L243) [36], anti-hHLA-DQ (clone Tu 169), anti-hCD4 (RPA-T4) (all from BD, Pharmingen, USA), and anti-mMHC class II (I-A/I-E) (eBioscience, USA). All samples were analyzed the day of sampling on a FACSCanto II (Becton Dickinson, USA).

### Immune function with KLH immunization

A functional immune response was verified by immunizing tgms and wt mice with KLH, a highly effective T-cell-dependent carrier protein that induces MHC class I and II restricted immune responses. Five DR7xhCD4 tgm and five wt mice were twice (14 days apart) intravenously immunized in the tail vein with KLH diluted in PBS (900μg/animal). Blood samples were taken from orbital plexus before first immunization and at the final bleeding five days after the second immunization. Serum was prepared and stored at -70°C until analyzed for anti-KLH specific IgG and IgM titres by ELISA (Life Diagnostics, USA) according to the manufacturer’s instructions. All samples were analyzed on the same day.

### Ximelagatran exposure of tgms and markers of liver injury

To study the ability of the tgms to mimic the hepatotoxic response seen in humans after ximelagatran exposure, wt and tg mice were dosed daily via gavage for 28 days with ximelagatran (120μmol/kg/day). The selected dose for the mice was chosen from the regulatory 28-day study performed with ximelagatran since it caused maximal pharmacologic effect without causing adverse bleeding effects. As an example, dosing to humans in one of the clinical trials was 24 mg (0.72μmol/kg/day) [37]. Ximelagatran administered orally is rapidly transformed to melagatran, its active form, and the bioavailability is 5 to 10% in rats and about 20% in humans with low between-subject variation [38,39]. The effect of tgm DR7 (not expressing hCD4) was used to investigate possible differences with and without presence of hCD4. Blood samples were collected the day before start of dosing (day -1), half way through the dosing (day 14), and at the termination of dosing (day 29) (Table 1). ALT levels in plasma were analyzed on the day of sampling (Cobas C 501, Roche Diagnostics, USA), and plasma samples for analysis of CSF1R, HMGB1 and GLDH were stored at -70°C and analyzed after the study. Presence of CSF1R in plasma was analyzed by ELISA. Briefly, plates were coated over night with anti-mouse CSF1R monoclonal antibody (R&D Systems, Abingdon, UK,2ug/ml). After washing and blocking, diluted samples (1/50) were added in duplicate and incubated (2h). Following washing, biotinylated anti-mouse CSF1R (R&D Systems) was added as a detection antibody. After incubation and washing, streptavidin/HRP conjugate and stop solution was added (R&D Systems, USA) and the plate was read using SpectramaxPlus microplate reader (450 nm) (Molecular Devices). All samples were analyzed on the same day. Recombinant mouse CSF1R (R&D Systems) was used as a positive control. Presence of GLDH in plasma was analyzed by ELISA according to the manufacturer (Nordic BioSite, Sweden, number EKM21150).

**Table 1:** Study design of ximelagatran exposure study.

Animal groups	Ximelagatran 120 μmol/kg/day	
Wild type	n = 10	(5 male, 5 female)
DR7xhCD4	n = 9	(5 male, 4 female)
DR7	n = 9	(4 male, 5 female)
DQ2xhCD4	n = 7	(4 male, 3 female)

Animals were dosed orally, once daily, with ximelagatran for 28 consecutive days. Blood samples were taken at day -1, 14, and 29.

Liver specimens from left and median lobe were collected at termination. HMGB1 expression was analyzed by RT-PCR using liver samples frozen in RNAlater^™^ (Qiagen, Santa Clarita, CA). mRNA was isolated using Rneasy Mini kit (Qiagen) and translated to cDNA using High Capacity cDNA RT kit (Applied biosystems, Foster City, CA). The samples were run on Applied Biosystems 7900HT (Applied Biosystems) using a standard protocol, and fold change (before vs. after treatment) was calculated. Gross liver pathology and histopathology was performed on all animals. The paraffin embedded tissue was stained with hematoxylin and eosin (HE stain) and periodic acid-Schiff (PAS).

### In silico modelling

**Fig 1:**
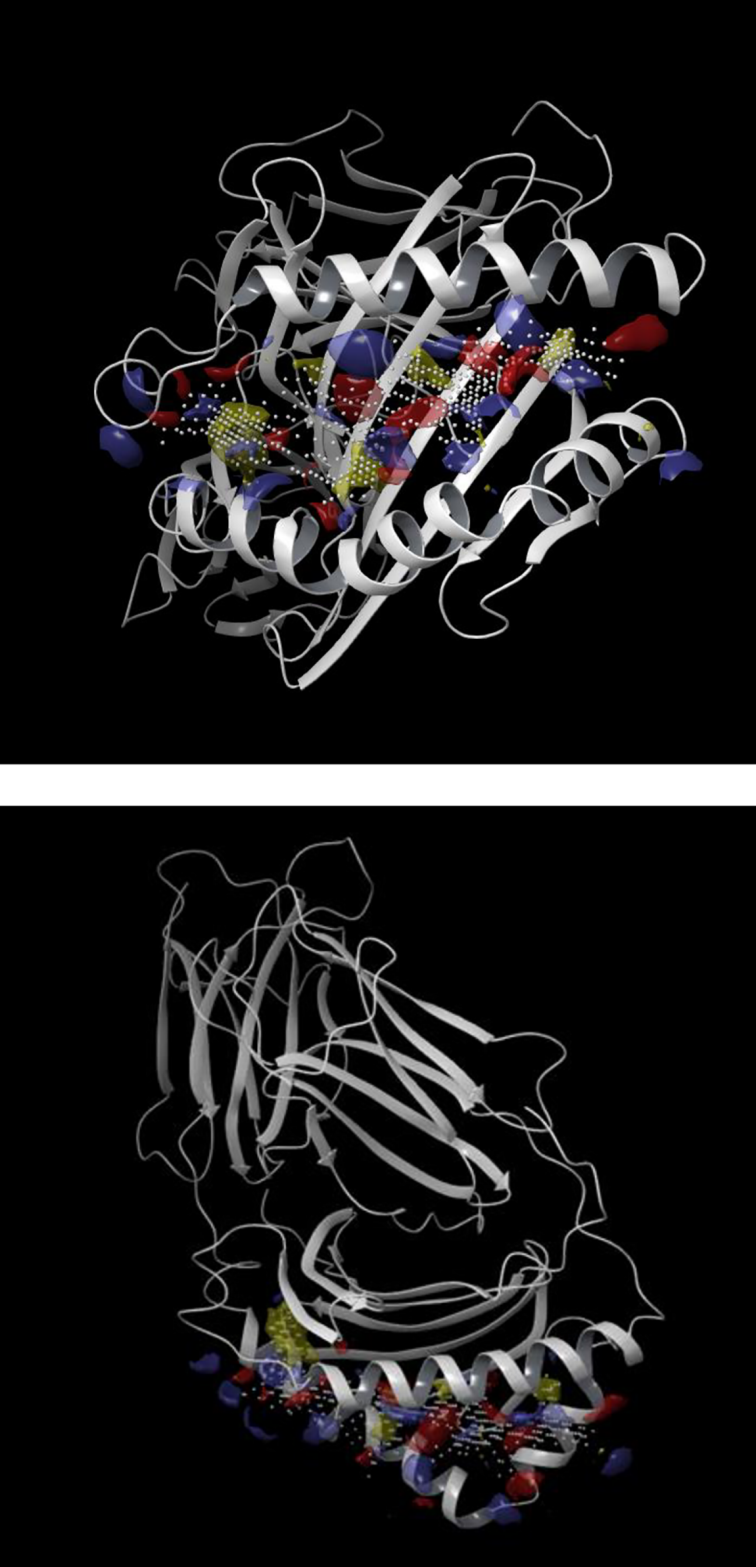
Identified antigen-binding groove binding-site. Antigen-binding groove binding-site identification of homology modelled HLA-DR7 using SiteMap (A–front view, B–top view). Blue–hydrogen bond donor region, red–hydrogen bond acceptor region, yellow–hydrophobic region, white–binding site grid.

Possible associations of ximelagatran (pro-drug) and melagatran (active drug) to the antigen-presenting groove of DR7 were investigated. First, the complete structure of HLA-DR7 was created using the UniProt BLAST algorithm [40], the identity was 92,1% between the β-chain of HLA-DR1 (UniProt ID: P04229) and HLA-DR7 (UniProt ID: P13761) after alignment. Due to this high sequence identity between the two chains, it was possible to create a HLA-DR7 model using the sequence of HLA-DR7 and an existing crystal structure of HLA-DR1 [41] with the homology modelling tool Prime [42]. Once the homology model of the HLA-DR7 β-chain was created, it was merged with the α-chain of HLA-DR1 structure to create a complete unit. Second, binding-site identification was performed on this complex using the SiteMap tool [43], with successfully identified the antigen-binding groove as a potential binding site (Fig 1).

With the information gathering from SiteMap, ligand-target molecular docking experiments were performed with Glide [44] using melagatran and ximelagatran as the ligands.

### Statistical analyses of ex vivo data

The nonparametric Kruskal-Wallis test was used for statistical comparisons between unmatched groups. If a significant difference between three or more groups was detected, Mann-Whitney test was used to compare the distributions of two unmatched groups.

## Results

### Characterization of the tgms

Phenotyping of lymphocytes and verification of HLA and hCD4 expression.

**Fig 2:**
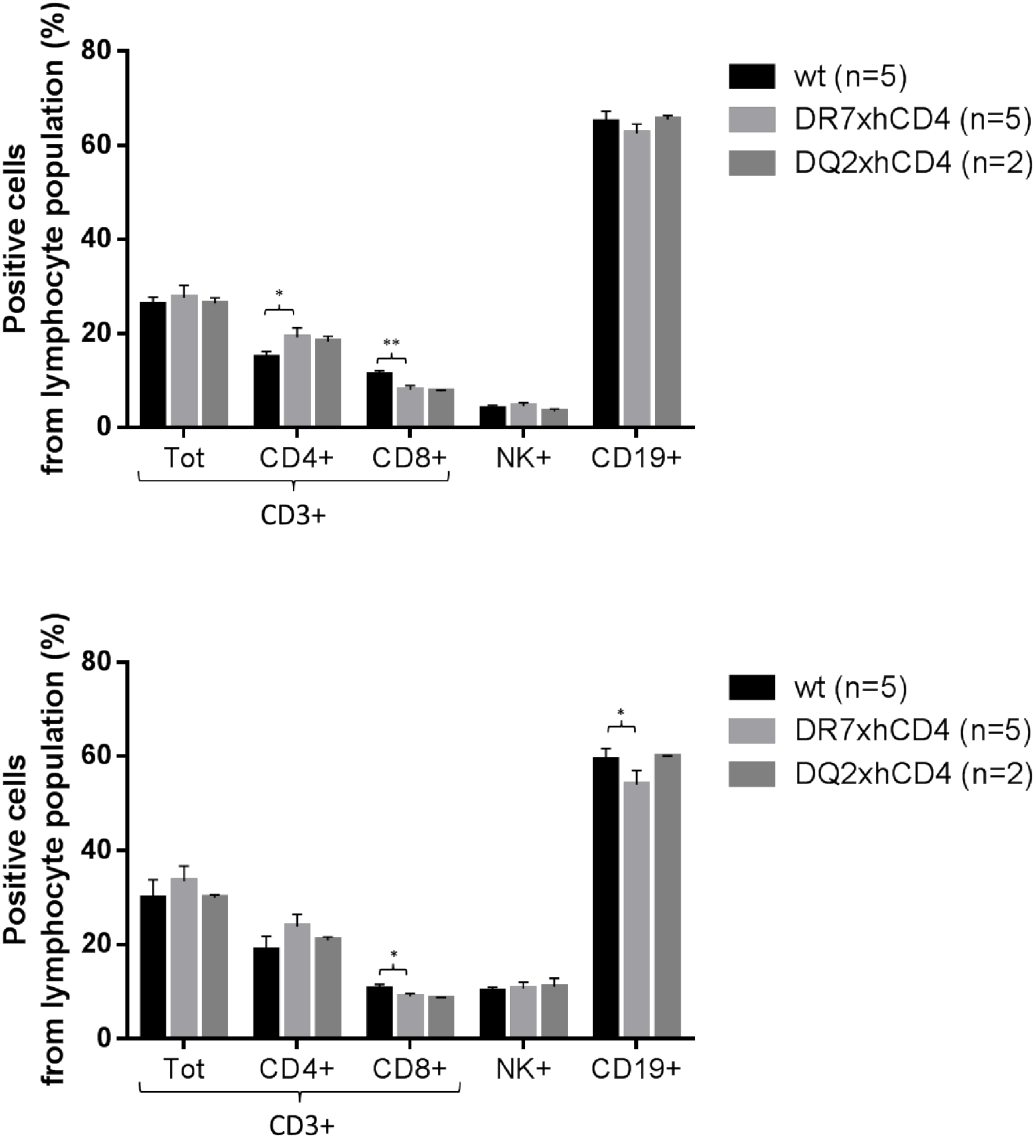
Verification of normal and comparable profiles between wt and tg mice. Representation of lymphoid cell subsets from PBMC (A) and spleen (B) to compare wt and tg mice using mouse specific tracer antibodies. *^/^** significant difference, * p≤0.05 ** p≤0.01.

**Fig 3:**
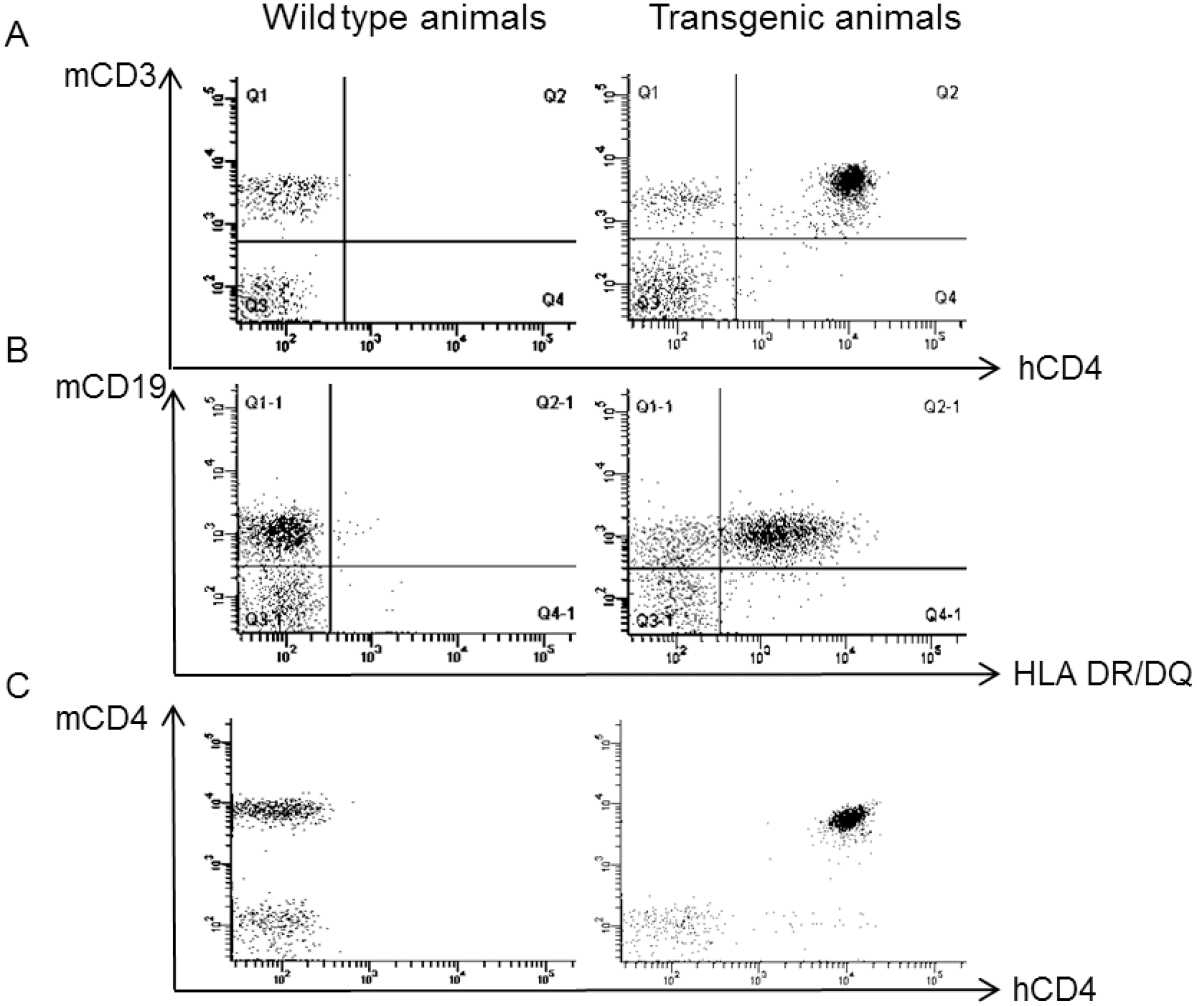
Representative surface-marker expression on cells from wt and tg mice. A and B illustrate the expression surface markers hCD4 and HLA DR/DQ on PBMCs, respectively. C displays the T-cell population and shows that hCD4 in the tgms is almost exclusively expressed on T-cells also expressing mCD4.

The proportion of lymphoid cell sub-populations from blood and spleen were compared between the tgms (DR7xhCD4 and DQ2xhCD4) and wt littermates to explore possible differences due to the insertion of human genes. The tgms showed similar proportions of total T- and NK-cell numbers compared to wt mice. However, significant differences between wt and tgms DR7xhCD4 mice could be seen; that is, DR7xhCD4 mice had fewer CD8+ T-cells in both spleen and PBMC, more mCD4+ T-cells in spleen, and fewer CD19+ cells in PBMC (Fig 2). The expression of hCD4 and HLA-DR/DQ on tgms compared to wt can be seen in Fig 3.

The introduction of hCD4 into the mouse genome resulted in significantly higher amount of hCD4+ T-cells in tgms compared with mCD4+ T-cells in wt mice in the PBMC population. In spleen population, the amount of hCD4+ T-cells in tgms was significantly lower than the mCD4+ T-cell population (Table 2). In PBMC of tgms, virtually all cells expressing mCD4 also expressed hCD4 (Fig 3).

**Table 2:** Surface markers.

			Wt		DR7xhCD4		DQ2xhCD4	
			% cells	SD	% cells	SD	% cells	SD
PBMC	T cell	mCD4 +	66.1	1.7	70.8*	3.0	68.7	3.2
		hCD4 +	0.1	0.0	74.0	3.1	70.4	2.5
	B cell	H2 +	99.3	0.3	98.6	0.8	98.9	0.1
		HLA +	0.9	0.1	59.9	3.0	52.4	3.1
Spleen	T cell	mCD4 +	60.9	3.3	61.4	4.8	61.7	8.1
		hCD4 +	2.0	2.5	53.5	3.5	56.6	3.6
	B cell	H2 +	99.0	0.2	97.9**	0.5	99.3	0.2
		HLA +	0.8	0.2	75.6	3.3	n.d.	

Percent of surface markers in the lymphoid cell populations from PBMC and spleen in tg- and wt-animals.

*/** significant difference compared to wt, * p≤0.05 ** p≤0.01

Tgms had fewer HLA+ B-cells compared to H2+ B-cells in wt mice. For DR7xhCD4 it was 40% (PBMC) and 23% (spleen) and for DQ2xhCD4 it was 47% (PBMC) fewer (Table 2).

### Immune function with KLH immunization

To verify normal *in vivo* immune function post HLA-insertion, strain DR7xhCD4 was immunized with KLH and the results compared to corresponding antibody responses in wt mice. All animals which were immunized with KLH responded with a distinct KLH-IgG and KLH-IgM specific response, and no statistical differences in titers of KLH-specific IgG- and IgM-responses were observed (S1 Fig).

### Ximelagatran exposure of tgms and markers of liver injury

**Fig 4:**
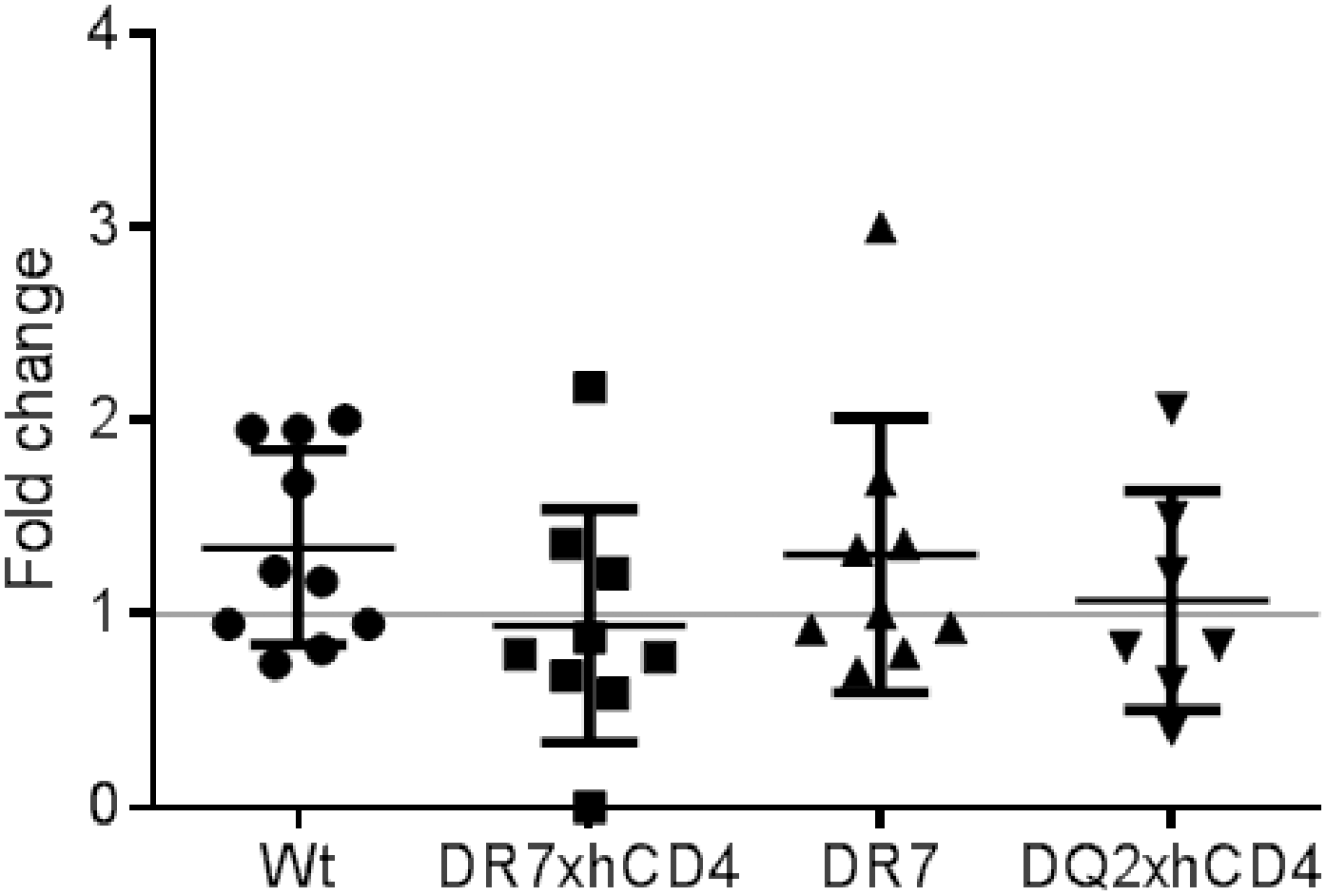
ALT levels in animals treated with ximelagatran for 28 days. ALT levels in mice compared before and after 28 days of ximelagatran treatment. A fold change of one equals no change between start of treatment and end of treatment; fold change of two equals two times higher ALT levels.

Daily oral ximelagatran exposure for 28 days showed no significant differences in group mean values of plasma ALT pre- and post-treatment in either wt mice or tgms (Fig 4). All values before and after treatment, with respect to sex and age, were within the 95% normal interval. Correspondingly, no significant differences were noted in group mean values of CSF1R levels between pre- and post-treated wt and tgms (S2 Fig). In addition, no significant difference was observed in mRNA expression of HMGB1 (S3 Fig) or levels of GLDH from plasma samples pre-study, day 14 and day 28 (S4 Fig) when comparing wt animals and tgms. Also, no significant difference was observed with regard to any macroscopic or histopathological changes in the liver of wt mice and tgms following ximelagatran treatment. No significant difference in any of the analyzed biomarkers could be when comparing tgms with or without hCD4 expression.

### In silico modelling

**Fig 5:**
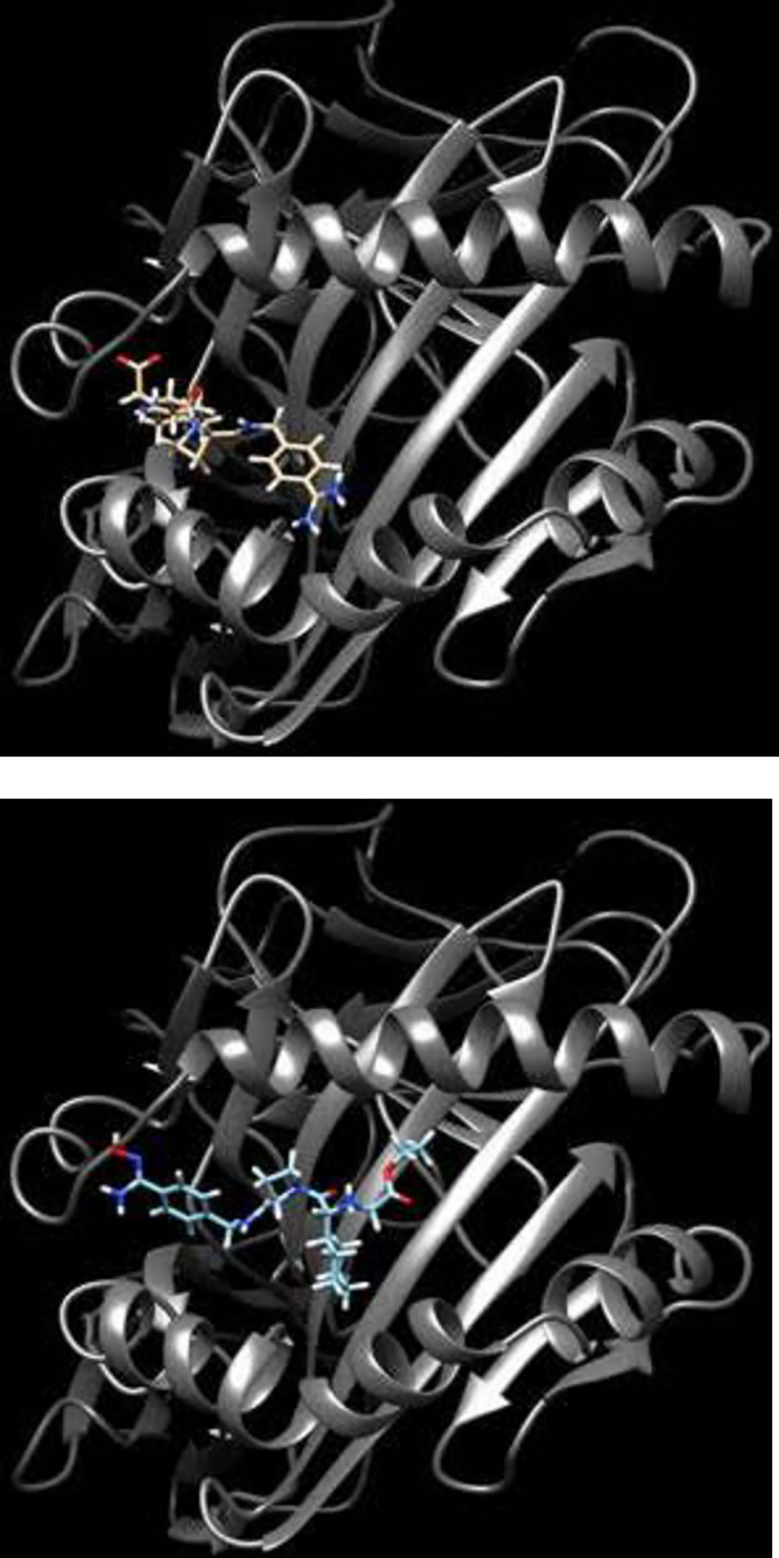
Structures of drug in HLA-DR7 model. Docked structure of melagatran (A–brown) and ximelagatran (B–blue) in the antigen-binding groove of the homology modelled HLA-DR7.

Both ximelagatran (pro-drug) and melagatran (active drug) are predicted to bind to the antigen-binding groove of HLA-DR7 in a similar location (Fig 5). However, the orientation of the best docked poses (from the docking score values calculated by Glide) differ between the two compounds. The reason behind this difference will be investigated in future in silico studies.

## Discussion

To our knowledge, this is the first study that evaluates whether HLA tgms can be used to predict DILI in humans following LMW drug treatment. Unlike many other HLA-transgenic models developed for use in the pharmaceutical industry [45–47], our system did not constitute a disease model for studying drug effects. Instead, we wanted to develop a safety model to investigate whether the tgms could imitate the liver effect seen in humans after ximelagatran treatment and be used in risk assessment.

Immunophenotyping of the lymphoid cell populations of produced tgms showed significant differences between wt and tgms. However, they were not considered to be of any biological relevance. Immunization with KLH did not indicate any significant difference in immune function between tgms and wt littermates.

For the first time, in silico-modelling studies revealed that both the pro-drug ximelagatran and the active drug melagatran have the capacity to associate to the antigen-binding groove of HLA-DR7. This new information importantly strengthens the hypothesis of these low-molecular entities having potential capacity to induce an “altered repertoire” driven immune response [15,25,28].

After ximelagatran treatment, no macroscopic or histopathological changes in the liver or signals from any of the tested biomarkers for liver injury (ALT, CSF1R, HMGB1, or GLDH) indicated any adverse liver reaction in either wt or tgms. Thus, none of our tgms responded to ximelagatran exposure as observed in humans exposed to ximelagatran.

Given our hypothesis that HLA-DR7 and/or HLA-DQ2 are necessary to initiate events leading to DILI from ximelagatran exposure, the sole presence of these receptors in another species was not enough to reproduce the adverse response observed in human. How can this be?

### Possible limitations for using HLA as a stand-alone denominator for addressing and predicting DILI

#### DILI development

The fact that DILI only develops in a small fraction of patients, even though the fraction is higher if patients carry the genetic susceptibility, makes the phenomena difficult to study. The number of animals in our studies could therefore have been too low to capture the development of DILI, even though individual differences between laboratory mice likely is less than between humans carrying a common genetic susceptibility marker.

#### HLA expression

The number of HLA positive cells and the HLA expression level could be an important factor and a possible limitation with our models. The expression of HLA-DR7 and HLA-DQ2 on B-cells was between 22 and 47% lower on tgms than the almost 100% expression of H2 on B-cells from wt mice. In humans, HLA-DR7 and HLA-DQ2 positive individuals express these alleles on all MHC-expressing cells. Thus, the relatively lower number of HLA-expressing cells could in our models reduce sensitivity to the drug. In our models the tgms still have the endogenous H2 complex intact. To amplify the contribution from HLA a H2 knockout mouse [48] could have been used to enhance the interaction between HLA and hCD4 with reduced possible interference of H2.

#### Genetic haplotype

Could the absence of DILI in our ximelagatran-treated tgms also be explained by the requirement of another genetic predisposition for developing DILI and could this be influenced by the haplotype involving genes other than HLA. One example is induced T-cell receptor repertoires playing an important part in autoimmune conditions [49]. Autoimmune hepatitis and DILI have common denominators and distinctions can be scarce. Ximelagatran-specific T-cell receptors from DILI-patients were not previously characterized and could therefore not be included in the current tgms.

#### Interaction between HLA and CD4

The sequence homology between human CD4 and mouse CD4 is about 80% for the intra cellular domain but only 55% for the extra cellular domain [50]. Both HLA-DQ [51,52] and HLA-DR [53] tgms can respond to specific antigens in an HLA-restricted manner both with and without hCD4. However, conceivably different HLA class II alleles or even different T-cell receptor/peptide/MHC complexes may have different CD4 requirements. Previous studies with HLA tgms have either used mice that are species-matched interaction with CD4 or mice that lack this interaction. Since both systems generate HLA-restricted responses [53,54], the importance of the requirement for species-matched CD4 remains unclear, even though we think a species-matched CD4 is an important factor for success.

#### Other conditions correlated to DILI

Conditions other than the presence of specific HLA may be needed for these models to predict DILI [55]. Earlier studies investigating the adverse effects seen after exposure to ximelagatran have proposed low nutritional status with low pyruvate levels [32], gender, and age [56] as potential risk factors for ximelagatran induced hepatotoxicity in humans. These three risk factors have been investigated in calorie restricted wt mice dosed with ximelagatran (Park BK et al, University of Liverpool, unpublished observation) without being able to establish a correlation.

Further, breaking of immune-tolerance in our model might have been yet another possibility to induce DILI by ximelagatran, allowing drug-specific lymphocytes to be activated. [57,58].

In conclusion, ximelagatran did not induce any signs of liver injury in any of the two tgms we constructed with the purpose of establishing a new safety model. Nevertheless, for the first time, the use of a HLA tg mouse model for prediction of HLA-associated DILI from a LMW drug has been evaluated. To particularly notice, for the first time we demonstrated that ximelagatran and melagatran are able to associate with HLA-DR7 obtained by in silico modelling.

## Supporting information

S1 Fig Levels of KLH specific antibodies. Expression of KLH specific IgG (A) and IgM (B) antibodies before and after immunization with KLH.
(PDF)

S2 Fig CSF1R in ximelagatran treated mice. CSF1R in plasma before (day-1), during (day 14), and after (day 29) 28 days of ximelagatran treatment.
(PDF)

S3 Fig Levels of HMGB1 mRNA expression before and after ximelagatran treatment for 28 days. Fold change of HMGB1 mRNA expression between start of treatment (day -1) and after end of treatment (day 29). A fold change of one demonstrate no treatment effect.
(PDF)

S4 Fig Levels of GLDH after 28 days of ximelagatran treatment. Levels of GLDH in serum samples from wild type (wt) and tgms (HLA-DRxhCD4 and HLA-DQxhCD4) mice. Samples are taken before (day -1), during (day 14), and after (day 29) 28 days of ximelagatran treatment.
(PDF)
