# Supplementary figures and images for: HLA-DR7 and HLA-DQ2: Transgenic mouse strains tested as a model system for ximelagatran hepatotoxicity
Source: PLoS One. 2017 Sep 21;12(9):e0184744. doi: 10.1371/journal.pone.0184744 (PMC5608249; doi:10.1371/journal.pone.0184744)

A

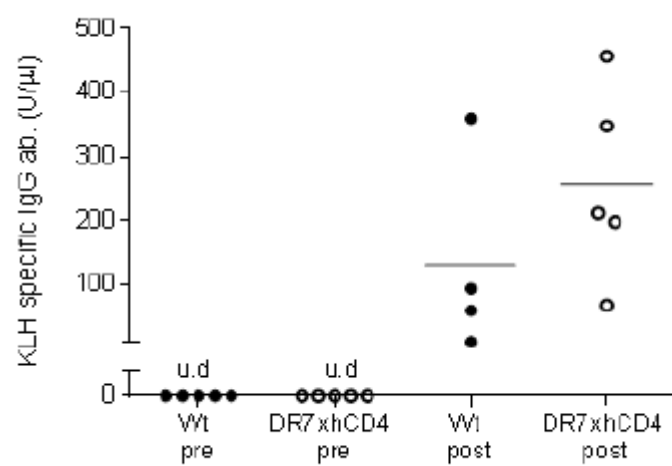

B

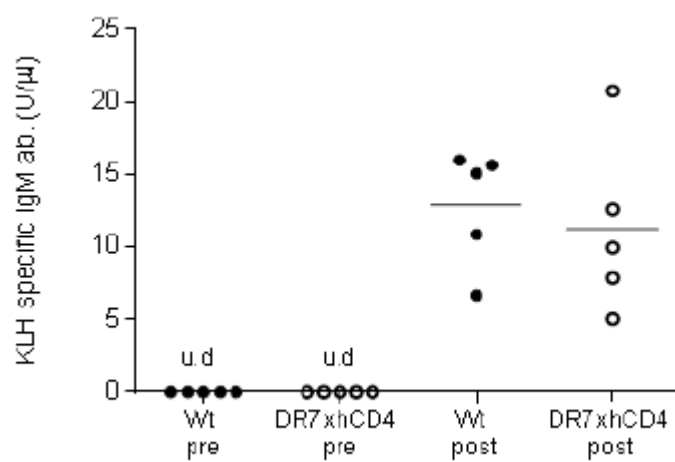

Supplement: S1 Fig — Expression of KLH specific IgG (A) and IgM (B) antibodies before and after immunization with KLH. (PDF) [file pone.0184744.s001.pdf]

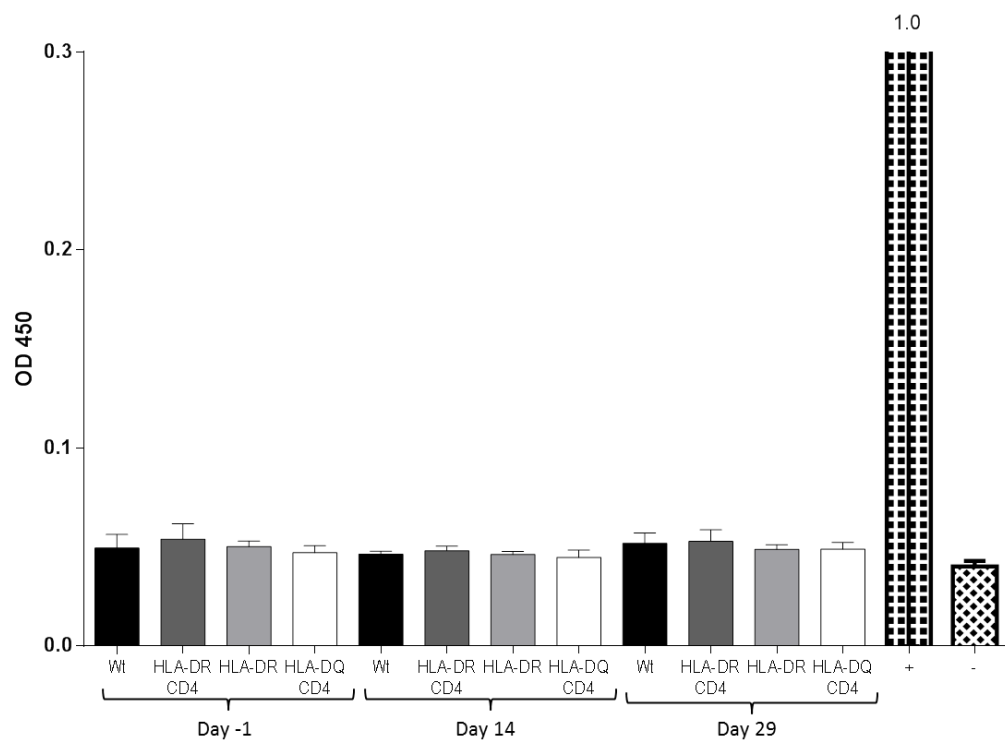

Supplement: S2 Fig — CSF1R in plasma before (day-1), during (day 14), and after (day 29) 28 days of ximelagatran treatment. (PDF) [file pone.0184744.s002.pdf]

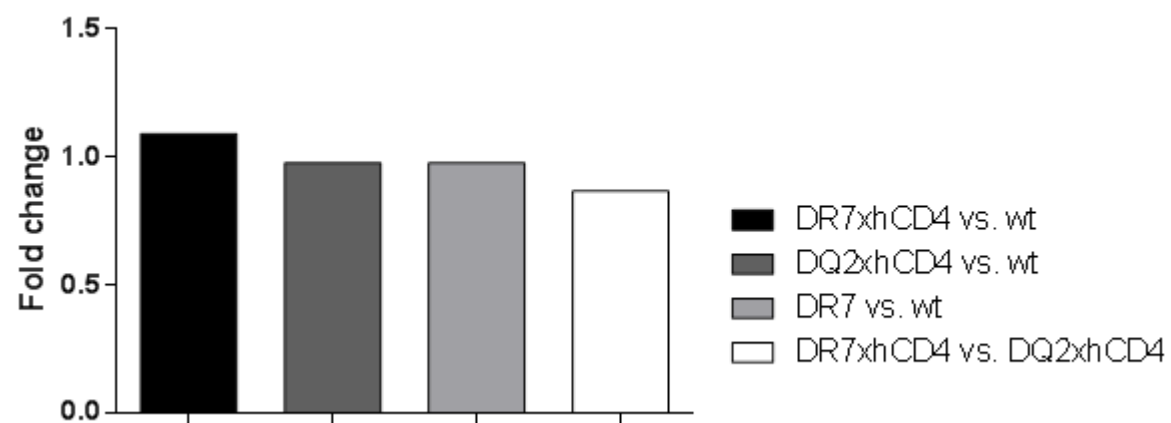

Supplement: S3 Fig — Fold change of HMGB1 mRNA expression between start of treatment (day -1) and after end of treatment (day 29). A fold change of one demonstrate no treatment effect. (PDF) [file pone.0184744.s003.pdf]

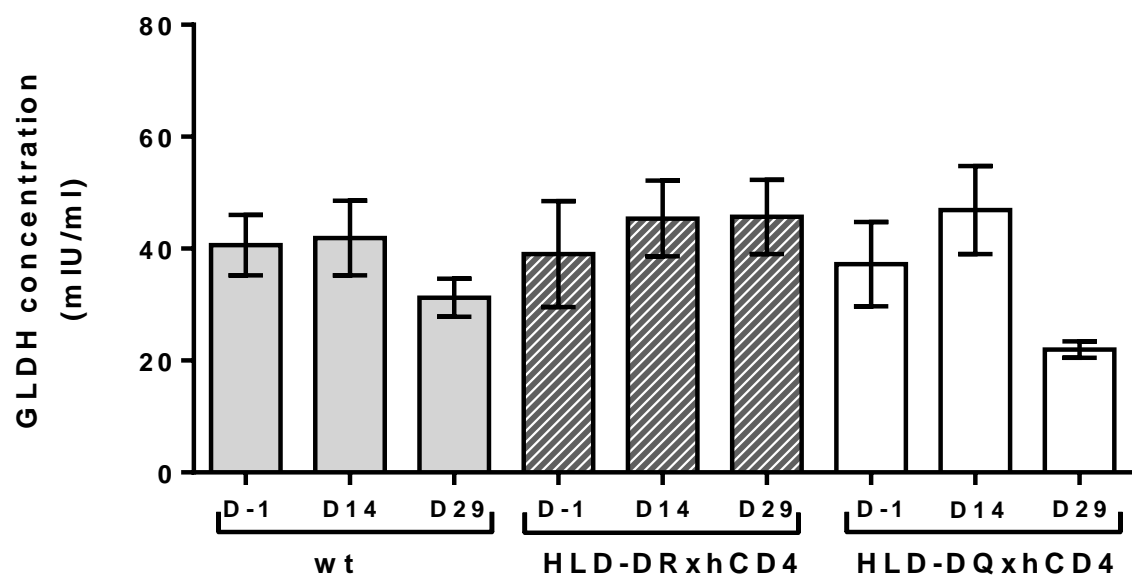

Supplement: S4 Fig — Levels of GLDH in serum samples from wild type (wt) and tgms (HLA-DRxhCD4 and HLA-DQxhCD4) mice. Samples are taken before (day -1), during (day 14), and after (day 29) 28 days of ximelagatran treatment. (PDF) [file pone.0184744.s004.pdf]
